# Can People Intentionally and Selectively Forget Prose Material?

**DOI:** 10.3389/fpsyg.2022.928533

**Published:** 2022-05-26

**Authors:** Bernhard Pastötter, Céline C. Haciahmet

**Affiliations:** Department of Psychology, University of Trier, Trier, Germany

**Keywords:** long-term memory, episodic memory, directed forgetting, selective forgetting, text memory

## Abstract

List-method directed forgetting (LMDF) is the demonstration that people can intentionally forget previously studied information when they are asked to forget what they have previously learned and remember new information instead. In addition, recent research demonstrated that people can selectively forget when cued to forget only a subset of the previously studied information. Both forms of forgetting are typically observed in recall tests, in which the to-be-forgotten and to-be-remembered information is tested independent of original cuing. Thereby, both LMDF and selective directed forgetting (SDF) have been studied mostly with unrelated item materials (e.g., word lists). The present study examined whether LMDF and SDF generalize to prose material. Participants learned three prose passages, which they were cued to remember or forget after the study of each passage. At the time of testing, participants were asked to recall the three prose passages regardless of original cuing. The results showed no significant differences in recall of the three lists as a function of cuing condition. The findings suggest that LMDF and SDF do not occur with prose material. Future research is needed to replicate and extend these findings with (other) complex and meaningful materials before drawing firm conclusions. If the null effect proves to be robust, this would have implications regarding the ecological validity and generalizability of current LMDF and SDF findings.

## Introduction

Memory research using the list-method directed forgetting (LMDF) task has shown that people can intentionally forget a previously studied list of items (e.g., words) when cued to do so. In a typical LMDF experiment, participants study two item lists. After studying list 1, participants are either asked to remember list 1 for an upcoming recall test (remember condition) or to forget list 1 because this list will not be tested (forget condition). After studying list 2, which is always to be remembered, participants are asked to recall both list 1 and list 2, independent of original cuing. Two effects typically emerge. First, forget-cued participants recall fewer list 1 items than remember-cued participants, which is referred to as list 1 forgetting. Second, forget-cued participants recall more list 2 items than remember-cued participants, which is referred to as list 2 enhancement (see Sahakyan et al., 2013, for a review).

Both inhibitory (i.e., retrieval inhibition; Geiselman et al., 1983) and non-inhibitory accounts (e.g., mental context change; Sahakyan and Kelley, 2002) have been put forth to explain the list 1 forgetting (see Bäuml et al., 2020, for a review). The retrieval inhibition account assumes that the forget cue triggers an active inhibitory control process that reduces the accessibility of the list 1 context representation, which leads to list 1 forgetting. The context change account, on the other hand, assumes that participants deliberately change the mental context in response to the forget cue (e.g., they think of something other than the experiment), which leads to an encoding/retrieval mismatch and thus to context-dependent forgetting of list 1. Regarding list 2, both accounts suppose that the reduced accessibility of the list 1 context reduces proactive interference for list 2 at the time of testing, which explains the list 2 enhancement. In addition, there is evidence that encoding factors can contribute to list 2 enhancement (see Sahakyan et al., 2013; Pastötter et al., 2017, for reviews).

In most LMDF studies, lists of unrelated words were used as item material (e.g., Pastötter et al., 2012, 2016; Abel et al., 2021). However, there are some exceptions. For instance, Barnier et al. (2007) demonstrated that people can intentionally forget autobiographical memories when these are generated by the participants in response to cue words and recalled together with the cue words at the time of testing. In addition, LMDF has been observed for product attributes (Shapiro et al., 2006), simple actions (Sahakyan and Foster, 2009), attitude statements (Waldum and Sahakyan, 2012), line drawings (Hupbach and Sahakyan, 2014), everyday objects (Hupbach et al., 2018), and behavioral descriptions (Scully and Hupbach, 2020). Thereby, the list 1 forgetting is typically eliminated when the items are related between lists (e.g., Conway et al., 2000; Barnier et al., 2007; Sahakyan and Goodmon, 2007). This is consistent with the view that the participants are reminded of the list 1 items during list 2 encoding, which can reinstate list 1 context and thus abolish list 1 forgetting. To our knowledge, no study has yet investigated whether intentional forgetting in the LMDF task arises for prose material. The present study closes this gap. Texts differ from unrelated study materials in several ways. They are usually meaningful and well structured. In addition, participants have more experience with texts and may find them more interesting than unrelated item materials (Kintsch, 1982). Therefore, the question of whether meaningful text can be intentionally forgotten or not addresses the ecological validity of current findings in LMDF research.

Furthermore, more recent research has demonstrated that people can selectively forget a subset of previously learned information when cued to do so. Selective directed forgetting (SDF) has been studied with three-list and two-list tasks. In the three-list task, participants study three lists of items and, after studying list 2, are either cued to remember both list 2 and list 1 (RRR condition), to forget list 2 but remember list 1 (RFR condition), or to forget both list 2 and list 1 (FFR condition). List 3 is always to-be-remembered. At test, participants are asked to recall the three lists’ items independent of original cuing. Lists 1 and 2 are tested first, counterbalanced in output order. Two outcomes are commonly observed. First, participants in the FFR condition recall fewer list 1 and 2 items compared to participants in the RRR condition, which indicates intentional forgetting. Second, participants in the RFR condition recall fewer list 2 items but the same number of list 1 items compared to participants in the RRR condition, which indicates SDF (Kliegl et al., 2013, 2018, 2020; Racsmány et al., 2019; Schmidt et al., 2021; but see Sahakyan, 2004). In addition, reproducible SDF has been demonstrated in the two-list task, in which, after studying list 1, participants are asked to selectively forget one subset of list 1 items and remember another subset of list 1 items (Delaney et al., 2009; Gómez-Ariza et al., 2013; Kliegl et al., 2013; Aguirre et al., 2017, 2020; Imbernón et al., 2022; but see Storm et al., 2013; Akan and Sahakyan, 2018).

SDF is thought to be based on inhibitory control (i.e., retrieval inhibition; Delaney et al., 2009; Kliegl et al., 2013, 2020), which is mediated in the prefrontal cortex (Imbernón et al., 2022). Indeed, the context change account cannot easily explain SDF. The effect was studied with unrelated words (e.g., Kliegl et al., 2013, 2020), short sentences (e.g., Delaney et al., 2009; Imbernón et al., 2022), and motor sequences of finger movements (Schmidt et al., 2021) as item material. No study has yet examined whether selective forgetting arises for unrelated prose passages. Both LMDF and SDF research has mostly been concerned with lists of unrelated items (e.g., words, sentences, and movements). Therefore, we know quite well under which conditions people are able to intentionally and selectively forget a list of unrelated words (e.g., regarding the role of post-cue encoding; Pastötter and Bäuml, 2007, 2010; Racsmány et al., 2019; Abel et al., 2021), but not about whether (or under what conditions) people can intentionally and selectively forget meaningful text passages. This study takes the first step in this direction.

Memory for meaningful text is superior to memory for unrelated word lists. As Kintsch (1982) put it: Participants who have been presented with a list containing 40 common words may recall about 10 words in a later test. In contrast, participants who read a meaningful text with 200 words for the same amount of time may recall about 100 of these words, although for the most part not verbatim. One important factor that contributes to the superiority of text memory is the schematic organization of the text information, represented in a highly associated hierarchical knowledge structure (Myers and O’Brien, 1998; Wolfe, 2005). This knowledge structure is built on distinct but highly associated levels of verbal and conceptual memory representations. On the conceptual level, the memories are both clustered and ordered, which significantly promotes retrieval efficiency for both the verbal and the conceptual memory representations at the recall test (Kintsch, 1982).

Whether the schematic organization structure of text prevents LMDF and SDF is currently not known. Studying LMDF with unrelated word lists, Bäuml and Samenieh (2010, 2012) argued that selective reprocessing (restudy or successful retrieval) of some of the to-be-forgotten list 1 items can spread activation to the other list 1 items and reactivate the inhibited list 1 context, thus reducing or even eliminating list 1 forgetting (see also Bäuml, 2019). Based on this view, LMDF and SDF may not be expected for meaningful prose material. Indeed, if text is represented in a highly associated hierarchical knowledge structure (Kintsch, 1982), the reactivation of some (verbal or conceptual) elements during retrieval should largely reactivate other (verbal and conceptual) elements, and thus eliminate LMDF and SDF.

## Materials and Methods

### Participants

The sample consisted of 126 participants (88 students, 38 non-students, 89 females, 37 males) with a mean age of 24.6 years (SD = 7.6 years; 18 to 56 years). All participants indicated that they speak fluent German (114 native speakers). They were tested individually or in pairs independently of each other and the study was conducted *via* the online platform Zoom. The required sample size was calculated with G*Power (v3.1.9.4; Faul et al., 2007). Given α=0.05 and desired power of 1−β=0.80 to detect a main effect of condition (RRR vs. RFR vs. FFR) with medium effect size (f=0.30; see Kliegl et al., 2020) in a one-way fixed-effect ANOVA, a minimal sample size of 111 participants was calculated.

### Material

The material consisted of three text passages, which were taken from Roediger and Marsh (2005) and translated into German. Each passage covered a topic from United States history (“Dorothea Dix”: 279 words; “Fallingwater”: 276 words, “Georgia O’Keefe”: 297 words). Each passage was equally often used in the three experimental conditions and served equally often as the first, second, and third studied text. *A priori*, 20 knowledge units were defined for each text passage. These units were used by the raters to assess participants’ memory performance in the three recall tests. The material is available at Open Science Framework.^1^

### Design

The experiment had a single-factor design with the between-subjects factor of cuing condition (RRR, RFR, FFR). In the RRR condition, both the study of text 1 and the study of text 2 were followed by a cue to remember the just studied text. In the RFR condition, the study of text 1 was followed by a cue to remember text 1, whereas the study of text 2 was followed by a cue to forget text 2. In the FFR condition, both the study of text 1 and the study of text 2 were followed by a cue to forget the just studied text.

### Procedure

Due to the COVID-19 pandemic, the study was conducted *via* Zoom in a synchronous manner (i.e., with real-time interaction between the experimenter and participants; see Archibald et al., 2019). Microsoft PowerPoint slides were used for the presentation of the informed consent, the instructions, the text passages, and the distractor. Google forms were used for response entry.

**Figure 1:**
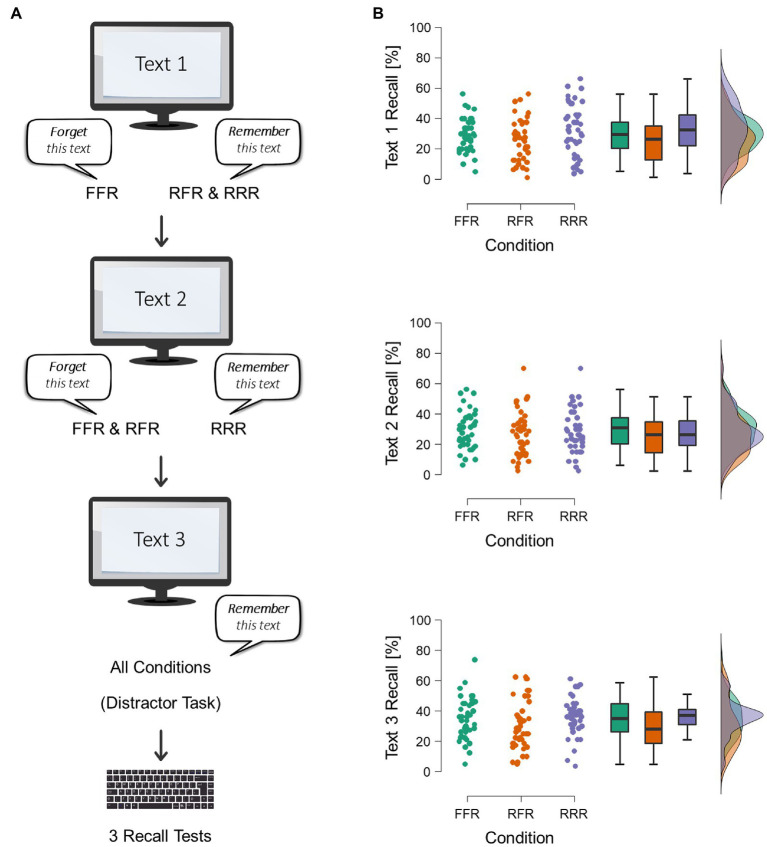
**(A)** Procedure. Participants were cued either to remember or to forget each text (text 1, text 2, text 3) immediately after the study of each text. A test, participants were asked to recall text 1 and text 2 first (testing order for the two lists was counterbalanced across participants) and text 3 last. **(B)** Recall results. Raincloud plots and boxplots for the recall data of text 1, text 2, and text 3. The results showed no significant differences between conditions (RRR vs. RFR vs. FFR).

Participants were told that they would be presented with three text passages about United States history facts and that they should learn these texts for later recall testing. In addition, participants were informed that following the presentation of each text, they would be given a cue to remember or forget the just studied text (see Pastötter and Bäuml, 2007, 2010; Kliegl et al., 2020). The experimenter emphasized that a to-be-forgotten passage would not be tested at later recall testing. Each text was presented on a single PowerPoint slide for 5 min. After the presentation of each text, participants were instructed either to remember or to forget the just studied text, depending on the cuing condition (see Figure 1A). After the presentation and cuing of text 3, participants did a spot-the-difference puzzle for 1 min as a distractor task. At test, participants were asked to recall the three texts irrespective of original cuing. Because the focus of this study was on intentional and selective forgetting of texts 1 and 2, output order was controlled and texts 1 and 2 were tested first. Half of the participants in each cuing condition recalled text 1 first and text 2 s, whereas for the other half of the participants the output order was reversed. Text 3 was always tested last. For each test, the title of the single passages (“Dorothea Dix,” “Fallingwater,” or “Georgia O’Keefe”) was provided and participants were asked to type what they could remember from each text in complete sentences using the keyboard. Recall time was 5 min for each text. After the recall of text 3, participants were thanked and fully debriefed.

### Data Analysis

Participants’ recall performance was assessed by two blind raters who were naive to the experimental paradigm and hypotheses as well as to the assignment of participants to experimental conditions. The two raters assessed participants’ recall performance based on the 20 pre-defined knowledge units for each text. The raters were instructed to give one point for each fully remembered knowledge unit and half a point for each partially remembered unit. The ratings were prepared independently by the two raters.

Intra-class correlation (ICC) estimates (ICC2.1 type as referenced by Shrout and Fleiss, 1979) and 95%confidence intervals (CIs) were calculated separately for the three recall ratings of text 1, $ICC=0.83,95\%CI\;\left[0.78,0.87\right]$, text 2, $ICC=0.86,95\%CI\;\left[0.82,0.90\right]$, and text 3, $ICC=0.75,95\%CI\;\left[0.68,0.81\right]$. These correlations suggest “excellent” (0.75−1.00) interrater reliability according to Cicchetti (1994). Therefore, the averaged ratings were used to analyze the recall data.

Both frequentist and Bayesian statistics were calculated. First, one-way ANOVAs with the factor of cuing condition (RRR vs. RFR vs. FFR) were calculated separately for text 1, text 2, and text 3. Levene’s test for homogeneity of variance was significant for both text 1 and text 3. Therefore, Welch homogeneity correction was applied in all further analyses. No significant main effects emerged in the ANOVAs; therefore, no post-hoc testing was required.

Second, Bayes factors (BFs) with r scale prior width 0.5 for fixed effects were calculated. $BF_{01}$ is reported for all analyses, indicating relative evidence for the null hypothesis compared to the alternative hypothesis (Wagenmakers et al., 2018). Jeffreys’s (1961) benchmarks were used to paraphrase the size of $BF_{01}$: anecdotal (1–3), substantial (3–10), strong (10–30), very strong (30–100), and decisive (>100) evidence for the null hypothesis compared to the alternative hypothesis.

All frequentist and Bayesian analyses were calculated with JASP (v 0.16.1; JASP Team, 2022). Raincloud plots and boxplots were also created with JASP. Data and analyses are available in a JASP file at Open Science Framework.^2^

## Results

### Text 1 Recall

Participants recalled on average 29.67% (SE=1.69%) of the text 1 units in the FFR condition, 25.57% (SE=2.11%) in the RFR condition, and 32.59% (SE=2.60%) in the RRR condition (see Figure 1B for visualization of the data). The one-way ANOVA with the factor of condition (RRR vs. RFR vs. FFR) showed no significant main effect of cuing, F(2,79.63)=2.34,MSE=304.15,p=.103. Bayesian analysis suggested anecdotal evidence for the null hypothesis, $BF_{01}=1.46$.

### Text 2 Recall

Participants recalled on average 29.88% (SE=1.89%) of the text 2 units in the FFR condition, 26.52% (SE=2.56%) in the RFR condition, and 28.27% (SE=2.14%) in the RRR condition (see Figure 1B). The one-way ANOVA with the factor of condition (RRR vs. RFR vs. FFR) showed no significant main effect of cuing, $F\left(2,81.54\right)=0.65,MSE=279.85,\;\;p=.525.$Bayesian analysis indicated substantial evidence for the null hypothesis, $BF_{01}=7.72$.

### Text 3 Recall

Participants recalled on average 34.94% (SE=2.09%) of the text 3 units in the FFR condition, 31.04% (SE=2.53%) in the RFR condition, and 36.16% (SE=1.87%) in the RRR condition (see Figure 1B). The one-way ANOVA with the factor of condition (RRR vs. RFR vs. FFR) showed no significant main effect of cuing, $F\left(2,80.87\right)=1.34,MSE=304.15,\;\;p=.268.$Bayesian analysis revealed substantial evidence for the null hypothesis, $BF_{01}=3.78$.

## Discussion

The results of this study suggest that LMDF and SDF do not arise for meaningful text. This is in contrast to earlier LMDF and SDF research showing that intentional and selective forgetting can be broadly present for different materials, including lists of unrelated words, short sentences, and simple actions. The present results are consistent with the view that text is represented in a hierarchical knowledge structure (Kintsch, 1982), which prevents LMDF and SDF. According to this view, reactivation (i.e., retrieval) of some of the to-be-forgotten (verbal or conceptual) elements of represented text broadly reactivates other elements of the text, which reactivates the whole text structure (or context), thus eliminating LMDF and SDF (Bäuml and Samenieh, 2010, 2012). In addition, the present findings with meaningful and relatively complex prose material may pose restrictions regarding the ecological validity and generalizability of previous LMDF and SDF research findings.

While most of the earlier LMDF research used item material that was unrelated both within and between lists, there is evidence from a study by Kimball and Bjork (2002) using Deese–Roediger–McDermott (DRM) word lists as item material that both list 1 forgetting and list 2 enhancement can arise for words that are semantically related within lists (see also Murphy and Castel, 2021, for a demonstration that item method directed forgetting can be significant but reduced for semantically related words compared to unassociated words). DRM lists, however, do not involve a hierarchical knowledge structure as is suggested for meaningful text. Knowledge structures are built on distinct but associated levels of verbal and conceptual representations. Importantly, on the conceptual level, the information can be meaningfully clustered and temporally ordered, which provides an efficient retrieval structure for the recall of both verbal and conceptual elements at the time of testing (Kintsch, 1982). In contrast, the single words in a DRM list are all associated with a single semantic associate, and within the list, the words are not further clustered or ordered, as is the case for the conceptual elements that are suggested to trigger the retrieval of the text.

While the results support the view that text is represented in a hierarchical knowledge structure (Kintsch, 1982), which prevents LMDF and SDF, the present study did not manipulate the meaningfulness of text and thus did not directly examine the role of schematic knowledge structures for LMDF and SDF of text. Future research is needed to address this issue. For example, Bransford and Johnson’s (1972) seminal work examined the influence of text comprehension on recall by manipulating whether or not participants were presented with a meaningful title (“washing clothes”) before studying a passage of text, which was rather incomprehensible without this title. Future research may like to use this approach to investigate the role of schematic knowledge structures for LMDF and SDF of text more directly. In addition, it needs to be shown to what extent the presentation of titles of the passages at the time of testing may have influenced the present results. The titles serve as cues to start the recall process and without the presentation of these titles, the inhibition of the to-be-forgotten passages might be preserved. In fact, this is what the study of Barnier et al. (2007) suggests, in which autobiographical memories were intentionally forgotten when the cue words were not provided at test.

Notably, in the present study, there was one procedural difference concerning the sequence of forget and remember cues in the FFR condition compared with previous SDF research (e.g., Kliegl et al., 2013, 2020). In the previous research, participants in the FFR condition were asked to remember list 1 after the study of list 1, but to forget list 1 after the study of list 2. In contrast, in the present study, participants in the FFR condition were cued to forget text 1 after they learned text 1 in the first place. Cuing sequences in the RRR and RFR conditions were identical to those in the previous research. We do not believe that this procedural difference in the FFR condition can explain the absence of LMDF effects in the present study. If anything, we would assume that providing the forget cue in the first place might increase the forgetting compared with presenting the forget cue after encoding the second list or text. Future research, however, may find it interesting to investigate this issue.

To conclude, findings from previous LMDF and SDF research have demonstrated that intentional and selective forgetting can arise for different kinds of material, including lists of unrelated words, short sentences, and simple actions. In contrast, the present study used relatively more complex, meaningful prose material and failed to observe significant LMDF and SDF effects. More future research using complex and meaningful materials (e.g., text, videos, and autobiographical memories; with or without titles or cue words provided at the time of testing) is needed to replicate and extend these findings before strong conclusions can be drawn. If it turns out that complex and meaningful materials are indeed not influenced by forget instructions, this could call into question the ecological validity and generalizability of current findings in LMDF and SDF research.

## Data Availability Statement

The datasets presented in this study can be found in online repositories. The names of the repository/repositories and accession number(s) can be found at: Material and data are available at Open Science Framework (OSF), https://osf.io/dakzn/, doi: 10.17605/OSF.IO/DAKZN.

## Ethics Statement

Ethical review and approval was not required for the study on human participants in accordance with the local legislation and institutional requirements. The patients/participants provided their written informed consent to participate in this study.

## Author Contributions

BP developed the study concept and experimental design, coordinated the data collection, analyzed the data, and drafted the manuscript. CH critically revised it. All authors contributed to the article and approved the submitted version.

## Funding

The publication was funded by the Open Access Fund of Universität Trier and the German Research Foundation (DFG) within the Open Access Publishing funding program.

## Conflict of Interest

The authors declare that the research was conducted in the absence of any commercial or financial relationships that could be construed as a potential conflict of interest.

## Publisher’s Note

All claims expressed in this article are solely those of the authors and do not necessarily represent those of their affiliated organizations, or those of the publisher, the editors and the reviewers. Any product that may be evaluated in this article, or claim that may be made by its manufacturer, is not guaranteed or endorsed by the publisher.

## Footnotes

^1^ https://osf.io/dakzn/

^2^ https://osf.io/dakzn/
